# Microvascular Environment Influences Brain Microvascular Heterogeneity: Relative Roles of Astrocytes and Oligodendrocytes for the EPCR Expression in the Brain Endothelium

**DOI:** 10.3390/ijms24086908

**Published:** 2023-04-07

**Authors:** Manjusha Thakar, Midrelle E. Noumbissi, Monique F. Stins

**Affiliations:** 1 Malaria Research Institute, Department Molecular Microbiology & Immunology, Johns Hopkins School Public Health, Baltimore, MD 21205, USA; 2 Walter Reed Army Institute of Research, Silver Spring, MD 20910, USA; 3 Biomedical Research Institute of Southern California, Oceanside, CA 92056, USA

**Keywords:** cerebral malaria, endothelial protein C receptor, blood–brain barrier, grey matter, white matter, astrocytes, oligodendrocytes, heterogeneity, cerebral blood vessels

## Abstract

Postmortem neuropathology shows clear regional differences in many brain diseases. For example, brains from cerebral malaria (CM) patients show more hemorrhagic punctae in the brain’s white matter (WM) than grey matter (GM). The underlying reason for these differential pathologies is unknown. Here, we assessed the effect of the vascular microenvironment on brain endothelial phenotype, focusing endothelial protein C receptor (EPCR). We demonstrate that the basal level of EPCR expression in cerebral microvessels is heterogeneous in the WM compared to the GM. We used in vitro brain endothelial cell cultures and showed that the upregulation of EPCR expression was associated with exposure to oligodendrocyte conditioned media (OCM) compared to astrocyte conditioned media (ACM). Our findings shed light on the origin of the heterogeneity of molecular phenotypes at the microvascular level and might help better understand the variation in pathology seen in CM and other neuropathologies associated with vasculature in various brain regions.

## 1. Introduction

The central nervous system (CNS) is a complex organ that needs strict homeostasis for optimal functioning. The blood–brain barrier (BBB) shields the brain from an influx of unwanted compounds but supplies it with needed nutrients and helps maintain homeostasis for the proper functioning of neurons and other brain cells. The cerebral endothelial cells (CECs) form the functional site of the BBB and display several features specific for the BBB endothelium, including an elaborate complex of tight junctions between adjacent CECs, a low transcytosis rate and the expression of specific membrane proteins on the luminal (blood facing) and/or abluminal (brain facing) membranes [1,2,3]. The brain is divided into different lobes and areas, including frontal cortex, parietal cortex and occipital cortex. The cortices, in turn, consist of two morphologically distinct tissues, grey matter (GM) and white matter (WM), which differ in terms of their cellular composition and vasculature [4].

In various neurological diseases and/or neuroinfections, very divergent neuropathologies are observed in different brain regions. Little is known about the underlying reasons for these differential pathologies in the brain’s GM verses the WM. In this study, we hypothesized that the neuropathological differences might be due to the heterogeneity of the brain microvasculature.

For example, in cerebral malaria (CM), a predominant WM pathology is observed, as evidenced by hemorrhagic punctae associated with an increased perivascular fibrin accumulation that is absent from the GM [3,5,6,7,8]. The vascular pathology in CM is a central hallmark caused by the sequestration of *Plasmodium falciparum*-infected red blood cells (PRBC) in the lumen of the brain’s microvasculature. The PRBCs bind to the brain endothelial cells via the parasite-encoded *P. falciparum* erythrocyte membrane protein-1 (Pf-EMP1) on infected PRBC. Among others, a host endothelial receptor for PRBC is the endothelial protein C receptor (EPCR) [9,10,11]. Because of its relevance in CM, we explored the expression of EPCR on microvessels derived from different areas of the brain and compared this with the endothelial cell markers CD31 and Glut-1. As in CM, the differential pathologies are clearly seen in areas such as the corpus callosum (CC) versus the frontal cortex (FWM/FGM) and the basal ganglia (BG); we focused on these areas and included vessels derived from the parietal lobe (PWM/PGM) and the occipital lobe (OWM/OGM). As the availability of human brain tissue is very limited, we performed this initial assessment of vascular heterogeneity with microvessels derived from bovine brains. To explore the underlying causes for heterogeneity, we followed up with in vitro experiments.

We found differences in the expression of EPCR on the microvessels, with generally higher levels in the WM regions compared to the GM regions. We hypothesized that the immediate brain environment of the microvessels, e.g., the relative abundance of astrocytes and or oligodendrocytes, would play a role in these molecular differences. Therefore, brain endothelial cells were cultured in vitro under conditions simulating the presence of endothelium in a WM versus GM brain environment; this resulted in a differential expression of EPCR. The inferences drawn from this study could help explain the differential neuropathologies, not only in CM, but could also broaden the scope to help understand other neurological diseases involving brain vascular pathologies.

## 2. Results

### 2.1. Heterogeneity in the Expression of EPCR on Cerebral Microvessels

**Figure 1:**
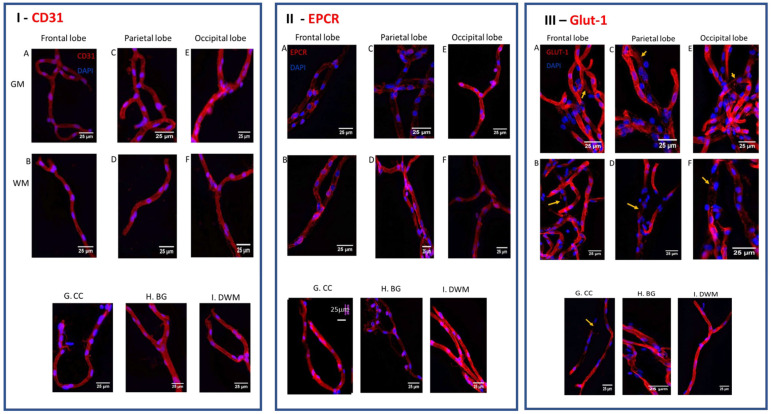
The heterogeneous expression of CD31, EPCR and CD31 on isolated bovine brain vessels. The expression of CD31 ((panel (**I**)), EPCR (panel (**II**)), and Glut-1 (panel (**III**)), was assessed on whole microvessels isolated from the different white matter (WM) and gray matter (GM) regions of the bovine brain, such as: corpus callosum (CC); basal ganglia (BG) and deep white matter (DWM). Red immunofluorescence showed a consistent presence of the endothelial marker CD31 on all vessels from all regions (**I**). The expression of EPCR (red color) was detected on microvessels from different regions but in relative different amounts; overall, more EPCR was expressed in vessels derived from white matter regions (**II**). Glut-1 expression showed on vessels of all regions but with varying intensity and in a mosaic pattern (**III**). Blue is DAPI staining, indicating nuclei. The pictures were taken at 40× magnification (bar = 25 micron).

The microvessels isolated from the various brain regions showed their typical “stringy” vascular morphology (Figure 1I–III). The relative expression of CD31, Glut-1 and EPCR, which was associated with these vessels from different regions of the brain, was visualized by immunofluorescence, as listed in the Materials and Methods Section. Their elongated morphology, size and presence of CD31 (Figure 1I) in the vessels from all regions confirmed their microvascular identity. Glut-1 expression (Figure 1III), another endothelial marker, was also confirmed, thereby corroborating the microvascular identity of the vessel preparations. Here, variances in the intensity of the immune labeling of the microvessels isolated from the different lobes were observed as well, even within one vessel. A mosaic pattern for Glut-1 labeling was clearly visible in the microvessels isolated from the WM of the parietal lobe, occipital lobe and corpus callosum (CC) (arrows) (Figure 1III).

The microvascular immune staining for EPCR (Figure 1II) was observed in all regions but with variable intensity. In the frontal lobes (F) and parietal lobes (P), the EPCR immune staining was somewhat higher in WM relative to GM; in the occipital lobe (O), the GM stained more strongly. The corpus callosum (CC) (Figure 1(IIG)), which consists mainly of WM, and deep white matter (DWM) (Figure 1III) showed substantially higher immune staining for EPCR than the basal ganglia (BG) (Figure 1(IIH)). This shows that, generally, there is a recognizable innate heterogeneity in the cerebral microvessels residing in the different brain lobes and in GM versus WM.

### 2.2. The Effect of Conditioned Media Derived from Oli-Neu and Astrocyte Cultures on the Expression of EPCR on Endothelial Cells

**Figure 2:**
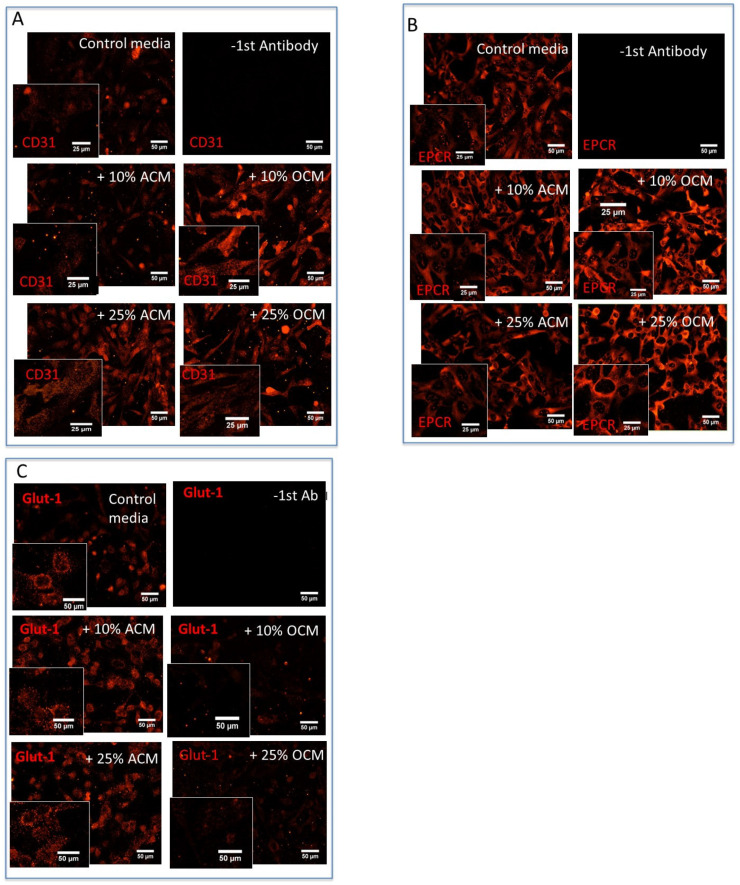
The expression of CD31 and EPCR in vitro on cultured HBEC-D3 cells. HBEC-D3 cells were grown in HBEC-D3 media with ACM or OCM media for 3 days before immunostaining with CD31 or EPCR. (**A**) Both ACM and OCM at 25% increased the CD31 expression on HBEC-D3. (**B**) ACM at 25% reduced the EPCR expression, whereas OCM increased the EPCR expression. (**C**) ACM affects Glut-1 expression but not OCM (M = 20× magnification, bar = 50 micron, Inserts: M = 40× magnification, bar = 25 micron).

**Figure 3:**
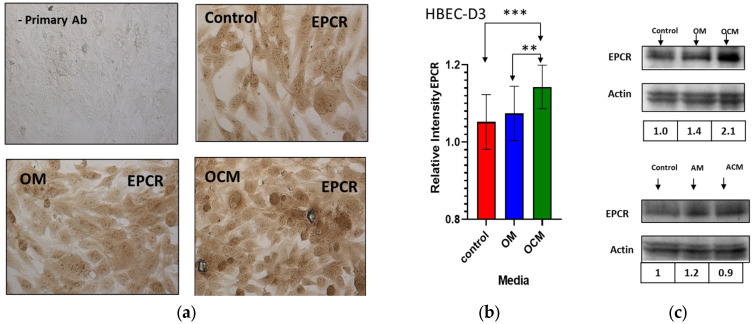
The effect of OCM media on the expression of EPCR in HBEC-D3. HBEC-D3s were grown in control media or in the presence of, respectively, OM or OCM media or ACM media with AM controls. (**a**) Immunostaining of HBEC-D3 with an anti-EPCR antibody (M = 40× magnification). (**b**) Quantification of IHC data by densitometry and analysis using ImageJ showed an increased expression of EPCR upon OCM incubation. (**c**) Western blot analysis for the EPCR expression on HBEC-D3 cells incubated with OCM or ACM. Bands were normalized to actin and showed a significant increase in the expression of EPCR upon OCM exposure. (**b**) The statistical analysis was carried out by one-way ANOVA with Tukey’s multiple comparison test (“**”, *p* ≤ 0.01, “***”, *p* ≤ 0.001).

In order to assess whether the immediate environment of the cerebral vessels influenced the EPCR expression, the in vitro culture conditions of the brain endothelial cultures were modulated. It is well publicized that astrocytes or conditioned media from astrocyte cultures confer a BBB phenotype in brain endothelial cells, which are usually derived from GM. Following a similar analogy, we hypothesized that oligodendrocytes could affect the phenotype of brain endothelial cells residing in WM and this could be responsible for the observed differences in EPCR expression. To test this, a conditioned medium was collected from both Oli-Neu cells (OCM) and from astrocytes (ACM). Brain endothelial HBEC-D3 cells were either incubated with the OCM or ACM media before assessing the expression of CD31 or EPCR by immunofluorescence (Figure 2A,B) and immunohistochemistry (IHC) (Figure 3a,b). The immunostaining of HBEC-D3 with an anti-CD31 antibody confirmed their endothelial derivation (Figure 2A). Irrespective of the inclusion of 10% or 25% of the OCM or the ACM in the regular endothelial growth media, the HBEC-D3 continued to present with the endothelial CD31 marker when grown in the presence of either kind of conditioned media. Interestingly, 10% ACM did not affect CD31 expression, but 25% ACM increased the immunolabeling intensity of CD31. Interestingly, 10% OCM and 25% OCM also increased the immunostaining of CD31 compared to the control HBEC-D3 growth media. Although ACM increased the Glut-1 expression on the HBEC-D3, the OCM did not have an effect on Glut-1 expression (Figure 2C). The control immune incubations without primary antibodies did not result in any background staining. This shows that the immediate environment of the microvessels, including astrocytes and oligodendrocytes, can specifically influence their functional molecular phenotype.

A unique observation was made as HBEC-D3 was grown with OCM consistently, thus showing a stronger EPCR immunostaining compared to the cells grown in the control media (Figure 2B). Both 10% and 25% OCM resulted in an increase in the EPCR immunostaining (Figure 2B), as did 50% OCM media. To further understand and quantify the effect of the OCM on EPCR expression, we compared this to the effect of non-conditioned oligodendrocyte media (OM) on the HBEC-D3 cultures. After incubation with the 50% OCM, 50% control OM and regular D3-growth media, endogenous levels of EPCR were assessed in the HBEC-D3 cells by IHC (Figure 3a). Additionally, here, an increase in the EPCR expression was observed after incubation with the OCM media. The OCM derived from murine oligodendrocytes and human oligodendrocyte–organoid cultures also increased the EPCR expression on HBEC-D3. This confirmed our earlier results of a visibly higher EPCR immunostaining upon OCM exposure compared to OM or control media.

Quantification of these results by densitometry analysis (Figure 3b) showed that the relative expression of EPCR consistently increased by about 10% upon OCM exposure relative to the control media (*p* ≤ 0.001). The effects of OCM and ACM on EPCR protein levels were also assessed in separate experiments using Western blot and densitometry (Figure 3c). Confirmation of equal loadings among all the samples was carried out by normalization to endogenous actin levels. Although no significant effect of ACM was found, here, OCM increased the EPCR band 2.0-fold times above the control media (Figure 3c).

### 2.3. The Effect of OCM and ACM Media on the Expression of EPCR on Endothelial and Macrovascular Cells

**Figure 4:**
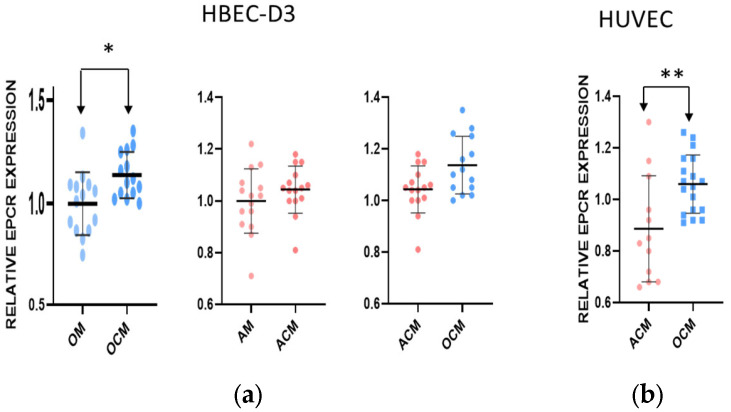
The effect of conditioned media on the EPCR expression in endothelial HBEC-D3 and primary HUVEC. (**a**,**b**) Endothelial HBEC-D3 cells and HUVEC macrovascular cells were grown in control media or in the presence of OCM or ACM, and the expression of EPCR was assessed. Quantification of EPCR by ELISA showed a differential expression of EPCR, with an increased expression after OCM exposure. Statistical analysis was carried out using a nonparametric unpaired *t* test (“*” *p* ≤ 0.05, “**” *p* ≤ 0.01).

To assess whether the effect of OCM on the expression of EPCR was specific for microvascular brain endothelial cells, we also tested this on macrovascular human umbilical vein endothelial cells (HUVECs) (Figure 4b). The expression of EPCR was quantified by ELISA. The effects of the ACM or OCM on the cells were compared to control non-conditioned AM and OM, respectively. This confirmed that the ACM did not have any significant effect on the expression of EPCR associated with the HBEC-D3. However, the expression of EPCR increased by about 12% after incubation with OCM, compared to ACM (Figure 4a) (*p* ≤ 0.05). The incubation of the HUVECs with OCM versus ACM resulted in a considerable increase in the expression of EPCR, by 22% (*p* ≤ 0.01) (Figure 4b). Thus, OCM positively affects the expression of EPCR on both macrovascular and microvascular endothelial cells; this effect is not specific to brain cells.

### 2.4. Heat Inactivation of OCM Eliminated Its Effect on EPCR Expression

**Figure 5:**
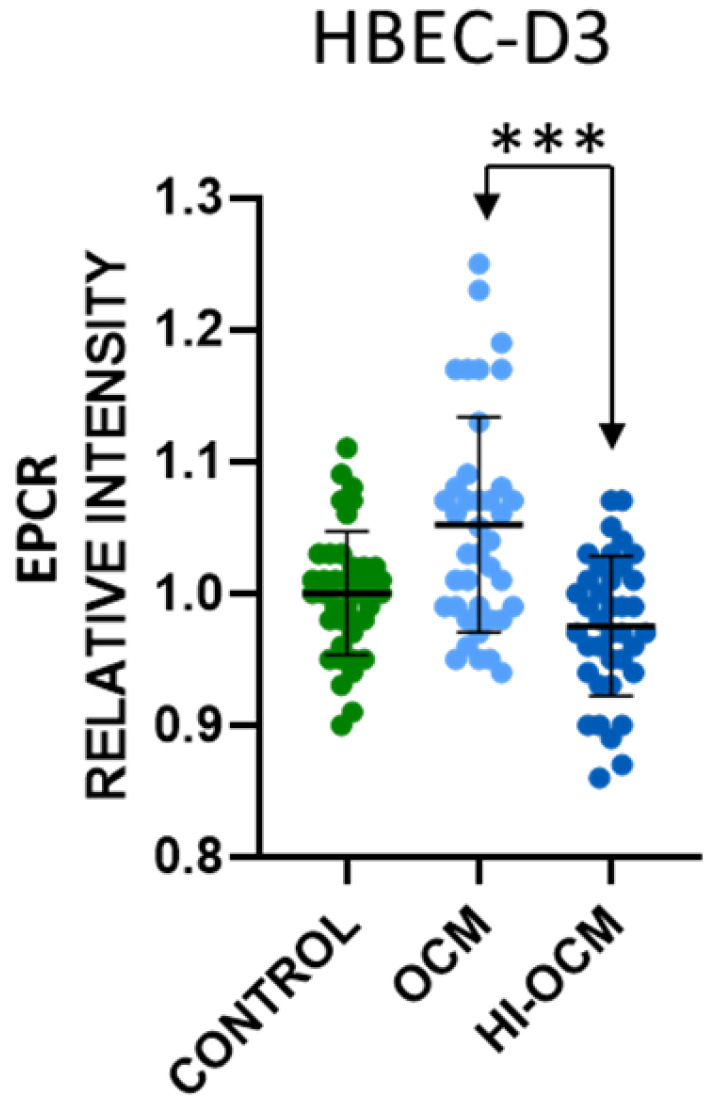
The effect of heat inactivation on conditioned media on the expression of EPC on in vitro cultured HBEC-D3 cells. HBEC-D3 cells were grown in OCM and heat-inactivated OCM and OM. The expression of EPCR was determined by IHC, quantified by densitometry and analyzed using ImageJ. Heat inactivation of OCM prior to incubation with the cells reduced the capacity to increase the expression of EPCR to control levels. Statistical analysis was carried out using one-way ANOVA with Tukey’s multiple comparison test (“***” *p* ≤ 0.001).

To assess whether the effect of OCM on the EPCR expression was due to a protein released by the oligodendrocytes, the OCM was heat-inactivated (HI) at 56 °C for 30 min. The effect of the HI-OCM was subsequently tested on the HBEC-D3, as carried out earlier (Figure 5). Heat inactivation of the OCM reduced the level of immunostaining of EPCR on the HBEC-D3 to control levels. Quantification confirms that the usual and consistent increase of 10% EPCR expression was abolished by heat inactivation (*p* ≤ 0.001) (Figure 5). This is suggestive of a protein-like compound in the OCM that modulates the effect on the endothelial cells.

## 3. Discussion

In this study, we report that a significant heterogeneity exists in the cerebral vasculature across various brain lobes and regions. Although this study initially started out from an interest in the sequestration of PRBC in CM pathology and the involvement of EPCR in this, it should be noted that EPCR also plays an important role in general organismal biology, including roles in the coagulation cascade, inflammatory signaling, endothelial barrier maintenance and cancer [12,13].

This overall heterogeneity was observed in whole microvessels preparations that were isolated from fresh bovine brain specimens. Bovine brain was used as human tissue, especially fresh tissue, is very difficult to source. Generally, more EPCR immunostaining was observed in vessel preparations isolated from WM than in those derived from GM areas, but with local differences. In addition, differences in immunostaining for EPCR were observed between frontal and parietal lobes of the brain. Furthermore, a strong EPCR signal was detected in vessels derived from the corpus callosum and a comparatively weaker expression was detected in the basal ganglia. These results signify a presence of basal heterogeneity in the expression of EPCR in various brain regions. We also observed differences in the intensities of immunostaining for both CD31 and Glut-1, with the latter showing a more mosaic-type pattern in some microvessels.

To assess whether the differential brain environments could have an influence on the microvascular phenotypes, we further investigated this by focusing on the expression of ECR on brain endothelial cells using in vitro cultures. Brain endothelial cells residing in a WM environment would then be more exposed to oligodendrocytes and their secretions, whereas those residing in a GM environment would be more exposed to astrocytes and their secretions. Astrocytes and their secretions are known to influence brain endothelial cell phenotypes [4] but little is known of the effects of oligodendrocytes. Analogue to this, a WM environment was simulated by including the conditioned media of Oli-Neu cells in the brain endothelial cultures. The Oli-Neu cells are readily available and have been shown to be a useful model for oligodendrocytes. Culturing the brain endothelial cells under these two different conditions, in the presence of ACM versus OCM, revealed that heterogeneity could be induced in in vitro endothelial cultures. The WM environment (OCM) up-regulates the expression of EPCR in the endothelial cells, whereas a GM cellular environment (ACM) down-regulates the expression of EPCR, thus creating a divergent expression. Although these findings are not specific to brain microvascular endothelial cells, as a similar effect is found with macrovascular HUVEC cells, they are specific for ACM versus OCM, as the expression of Glut-1 was not affected by OCM. Although we did not observe large differences, they were statistically significant and show proof of concept that, like astrocytes, oligodendrocytes influence the receptor expression on the endothelium of the brain vasculature.

We do not make any claims that the OCM and ACM fully represent a WM or GM environment, but merely demonstrate divergent effect of the immediate microvascular environment on the BBB phenotype. Other brain cells, including microglia and pericytes, also influence the brain endothelial phenotypes. As reviewed by Villabona et al. [4], many studies showed effects of astrocytes and/or their conditioned media on brain endothelial phenotypes, including increasing junctional molecules and the expression of transporters. Other studies have highlighted the effects of pericytes on the brain endothelial phenotype [14]. Together, these studies led to the concept of the neurovascular unit. However, very little is known of the effects of oligodendrocytes on the brain endothelial phenotype. The neurovascular unit has a very different composition in the GM than in the WM, and the metabolic needs of the underlying tissues are vastly different. Therefore, it is conceivable that the endothelium would also be differentially expressing various receptors, including the EPCR or Glut-1. Since at the cellular level, the immediate environment of the cerebral blood vessels is highly heterogeneous, these interactions and their effects may differ along the vessels, creating a patchwork of the expression of specific receptors. This would also explain the patch-like appearance of the CD31 and Glut-1 immunostaining in the microvessels, as we found in this study. More studies are needed to further unravel these differences and the additional effects of the GM environment versus WM environment on the brain’s vasculature.

These molecular differences are in addition to the known differences in the vascular distribution in GM and WM. For example, the GM vessels are more arranged perpendicularly in the brain parenchyma whereas the WM has fewer vessels, which are arranged more parallel to axons; in addition, more coiling of microvessels is observed in the WM [15,16]. The matter that the direct vascular environments do matter has been reported previously for different organs, as well as the differences between large vessels and microvessels, arteries and veins [17]. Vascular heterogeneity has also been shown along the vascular tree [18,19], including the differential expression of alkaline phosphatase, TNAP and ecto-phosphatase [20,21,22]. This includes the expression of EPCR, as this is higher in large vessels and lower in microvessels [23], which we noticed as well. More recently, Hase et al. [24] reported that besides the differences in microvascular densities between WM and GM, these vessels also responded differentially in dementia and stroke. More research is needed as to why some diseases exhibit differential pathologies in the brain’s GM and WM, and thus could be due to differential cerebral phenotypes, leading to heterogeneous responses. In CM, differential pathology is clearly observed in WM versus GM, with a clear abundance in hemorrhagic punctae in WM. CM pathology is, in part, associated with the sequestration of PRBC on host receptors such as ICAM-1 and EPCR. Since the relative abundance of these receptors differs under inflammatory conditions and in various brain regions, this not only influences the potential amount of sequestration but also impacts the host endothelial signaling responses in these vessels, leading to the differential pathologies in WM. Despite the higher vascular density in GM, in CM, more hemorrhagic punctae can be observed in the WM [25].

Other conditions, including vascular dementias, also include vascular components associated with WM hyperintensities, but at the molecular level, the causes are yet unclear [26]. A review by Lin et al. [27] shows that multiple factors may contribute to the WM pathologies, including segmental arteriolar disorganizations, the differential expression of collagenases and BBB disruptions associated with WM lesions. Stroke-prone rats also showed more WM damage preceding subcortical ischemic changes. All these factors are indicative of vascular heterogeneity, but more attention to the applications of gene technologies in order to clarify such differences could be given. Genome-wide linkage studies have predominantly focused on whole brain analysis and revealed a number of WM-related genes that contribute to differential pathologies [27], but the specific vascular components in these studies were not addressed and need further study.

The concept of endothelial heterogeneity is becoming more and more prevalent in the analysis and understanding of the brain vascular networks [1]. The exact nature of the factors underlying the observed heterogeneity is unclear and is likely multifactorial, but molecular communications between the brain endothelium and pericytes, astrocytes and oligodendrocytes could be the key. We attempted to obtain a bit more clarity on the nature of the component in the media responsible for the upregulation of EPCR. The fact that the heat inactivation of OCM consistently prevented an upregulation of EPCR is suggestive of the fact that the component involved is a protein. In addition, freeze/thaw cycles or extended storage at 4 °C diminished its ability to affect EPCR expression. Moreover, the maturity and density of the oligodendrocyte cultures contributed to the variance of the results, suggesting that the released factor(s) are also dependent of the growth stage of oligodendrocytes. Besides its effects on the expression of EPCR, other receptors and/or transporters may also be affected by factors released from neighboring oligodendrocytes.

A better comprehension of the cerebral vascular heterogeneity will help explain region-dependent regulatory mechanisms that influence the diverse BBB phenotypes. Understanding brain vascular heterogeneity is not only of importance for understanding diverse neuropathologies but is also significant for drug design and targeting specific affected areas.

## 4. Materials and Methods

### 4.1. Materials

Immortalized human brain endothelial cells (HBEC-D3) were a kind gift from Dr Weksler (Cornell University, Ithaca, NY, USA) [28]. Pooled human umbilical vein endothelial cells (HUVEC) were purchased from LONZA (Cat# cc-2519, Basel, Switzerland). Fresh bovine brain was purchased from a local abattoir (Oella, MD, USA). Oli-Neu cells were a kind gift from Dr. Trotter (University of Mainz, Mainz, Germany) [29]. The human astrocytes were isolated from surgical resections obtained from the Hopkins Brain bank and approved by the Johns Hopkins Institutional Review Board. The following antibodies were purchased as follows: anti-EPCR was from Invitrogen (Cat# PA5-12519 and PA5-79882, Waltham, MA, USA) and Abcam (Cat# ab56689, Cambridge, UK), biotinylated anti-rabbit and anti-mouse were from Vector Laboratories Inc. (Cat# BA-1000 and BA-9200, Newark, CA, USA, respectively), anti-CD31 was from ThermoFisher Scientific (Cat# MA3100, Waltham, MA, USA) and R&D systems (Cat# BBA7, Minneapolis, MN, USA), anti-Glut-1 (cat# PA1-1063) was from Thermofisher Scientific, horse radish peroxidase (HRP) coupled with anti-rabbit antibody was from cell signaling (Cat#7074s) and fluorescently tagged secondary antibodies were from Invitrogen (Cat# A31570 and A11012). The chamber slides were from Nunc (Cat#154534, Rochester, NY, USA). The Vectastain Avidin-Biotin Complex alkaline phosphatase (ABC-AP) kit (Cat# AK-5000) and Vectastain Elite ABC-Peroxidase (ABC-PO) kit (Cat# PK-6100) were from Vector Laboratories Inc.

### 4.2. Cell Cultures and Conditioned Media Collection

All cells were grown at a temperature of 37 degrees Celsius and in the presence of 5% CO_2_ in a humid atmosphere. HBEC-D3 cells were grown in 5% fetal bovine serum, 1% penicillin-streptomycin, 5 µg of ascorbic acid, 1/100 chemically defined lipid concentrate, 10 mM HEPES and 1 ng of basic fibroblast growth factor (bFGF) in EBM-2 media as described previously [28,30]. Human umbilical vein endothelial cells (HUVEC) were grown in the endothelial cell growth media (EGM-2) as per the manufacturer’s instructions. Oli-Neu cells were grown in Dulbecco’s modified Eagles media (DMEM) containing Sato supplement, penicillin-streptomycin, 1 mM of sodium pyruvate, 5 µg/mL of insulin, 5 µg/mL of N-Acetyl-1-cysteine and 10 ng/mL of d-Biotin and Trace elements B, as previously described [29,31]. Human astrocytes were grown in a minimum essential media (MEM) containing 10% fetal bovine serum and penicillin-streptomycin. Conditioned media from Oli-Neu cells (OCM) and human astrocyte (ACM) cultures were collected at 50–80% confluence every day for 4 days and replaced by fresh media. The conditioned media was centrifuged and supernatant stored at −20 °C and used in the experiments. Control media, not incubated with either Oli-Neu cells or astrocytes were, respectively, oligodendrocyte media (OM) and astrocyte media (AM).

### 4.3. Brain Vessel Isolations

The availability of human brain tissue for research studies is highly limited; therefore, bovine brain tissue was used. Whole brain microvessels were isolated from different lobes and regions of freshly obtained bovine brains: BG, CC, DWM, FGM, FWM, PGM, PWM, OGM and OWM. Each brain tissue region was dissected, homogenized and subjected to 15% high molecular weight dextran density gradient centrifugation at 10,000× *g* for 10 min at 4 °C, as previously published [32]. The pelleted vessels were washed and subsequently fixed in 4% paraformaldehyde in phosphate-buffered saline (PBS) or, as needed, stored as a non-fixed pellet at −80 °C.

### 4.4. Immunohistochemistry and Immunofluorescence

#### 4.4.1. Microvessels

The paraformaldehyde-fixed cerebral vessels were stained with the indicated primary antibody followed by a fluorescently tagged secondary antibody using a standard immunostaining protocol. Vessels were visualized and representative pictures and z-stacks were taken with the same exposures using a Nikon i90 microscope equipped with Velocity software v3.8. Subsequently, image z-stacks were deconvoluted with Velocity software.

#### 4.4.2. In Vitro Cell Cultures

HBEC-D3 or HUVECs (20,000 per well) were plated in collagen-coated chamber slides. The cells were incubated overnight at 37 °C and 5% CO_2_ before being exposed to either 25–50% of OCM or ACM media. The control Oli-Neu media (OM) or astrocyte media (AM), which had not been incubated with the cells, was included at the same concentrations. Every day, cells were replenished with fresh conditioned media for exactly three days. At the end of the treatment, cells were fixed with 50% acetone/methanol for 10 min, air-dried and stored at −20 °C. Subsequently, standard immunohistochemistry (IHC) was carried out using the Vectastain ABC-PO protocol. To reduce background endogenous peroxidase (PO) staining, cells were first quenched with 0.3% hydrogen peroxide for 10 min followed by a blocking step with 10% normal serum and subsequently with primary and secondary PO-coupled antibodies, as described previously [33]. The PO tags were visualized using a diaminobenzidine substrate. For fluorescence staining, commercially available fluorescently tagged secondary antibodies were used, as indicated in the results. The images were taken using a Zeiss Axiovert microscope with an AxioCam MRc05 camera combined with AxioVision v4.8. software. The staining intensity of the cellular surfaces was quantified using ImageJ. The data were analyzed by normalizing each data point to the control media alone and then were plotted using Graph Pad prism 8.0.

### 4.5. Quantification by ELISA

HBEC-D3 or HUVECs (20,000 per well) were plated in collagen-coated 96-well plates. The cells were incubated and subjected to a similar incubation protocol with either OCM or ACM, as outlined above. After incubation, cells were fixed with acetone: methanol (1:1), air-dried and stored at −20 °C. Cells were blocked with 10% normal serum and incubated with the indicated primary antibody followed by ABC-AP incubations, as published for ICAM-1, but with modifications for cell-associated EPCR [34]. The alkaline phosphatase tags were developed with p-nitrophenyl phosphate substrate in the presence of levamisole for 30 min, resulting in a soluble product. The optical density (OD) was read on a POLAR star OPTIMA plate reader at 405 nm. The data were normalized to the respective control media and analyzed using GraphPad Prism 8.0.

### 4.6. Western Blotting

The cells or vessels were lysed with lysis buffer (Cell Signaling, Cat# 9803, Danvers, MA, USA) using a standard protocol wherein the lysate was tumbled for 30 min at 4 °C followed by centrifugation at 18,000× *g* in a tabletop microfuge for 30 min at 4 °C. The protein concentration was determined by the standard bicinchoninic acid (BCA) method and 60 µg of protein per lane was loaded onto 4–12% Tris-glycine gels. The gel was run at 135 V. After running the gel, the protein bands were transferred onto nitrocellulose, blocked with 5% bovine serum albumin and incubated with a primary antibody followed by a secondary HRP-tagged antibody before developing with an electro-chemiluminescence detection reagent. The bands were analyzed using ImageJ software v1.53.

### 4.7. Statistical Analysis

The statistical analyses were carried out as indicated in the respective figure legends by either a one-way ANOVA with Tukey’s multiple comparison test or an unpaired *t* test.

## 5. Conclusions

The brain vasculature is heterogeneous, in several respects, with a differential expression of receptors, not only along the vascular tree as earlier reported, but also in different brain areas, as demonstrated here for EPCR and Glut-1. The direct cellular environment of the microvessels in either the WM and GM brain areas, respectively, including oligodendrocytes and/or astrocytes, contributes to the differential expression of receptors such as EPCR. In addition, other receptors and proteins are likely to be modulated on a local microvascular level as well; this is also relevant to the diversity in neuropathologies and to drug targeting.

## Data Availability

No genomic date were obtained for this study.

## References

[1] Nyul-Toth A., Suciu M., Molnar J., Fazakas C., Hasko J., Herman H., Farkas A.E., Kaszaki J., Hermenean A., Wilhelm I. (2016). Differences in the molecular structure of the blood-brain barrier in the cerebral cortex and white matter: An in silico, in vitro, and ex vivo study. Am. J. Physiol. Heart Circ. Physiol..

[2] Sofroniew M.V., Vinters H.V. (2010). Astrocytes: Biology and pathology. Acta Neuropathol..

[3] Dorovini-Zis K., Schmidt K., Huynh H., Fu W., Whitten R.O., Milner D., Kamiza S., Molyneux M., Taylor T.E. (2011). The neuropathology of fatal cerebral malaria in malawian children. Am. J. Pathol..

[4] Villabona-Rueda A., Erice C., Pardo C.A., Stins M.F. (2019). The Evolving Concept of the Blood Brain Barrier (BBB): From a Single Static Barrier to a Heterogeneous and Dynamic Relay Center. Front. Cell. Neurosci..

[5] MacPherson G.G., Warrell M.J., White N.J., Looareesuwan S., Warrell D.A. (1985). Human cerebral malaria. A quantitative ultrastructural analysis of parasitized erythrocyte sequestration. Am. J. Pathol..

[6] Warrell D.A. (1997). Cerebral malaria: Clinical features, pathophysiology and treatment. Ann. Trop. Med. Parasitol..

[7] Ponsford M.J., Medana I.M., Prapansilp P., Hien T.T., Lee S.J., Dondorp A.M., Esiri M.M., Day N.P., White N.J., Turner G.D. (2012). Sequestration and microvascular congestion are associated with coma in human cerebral malaria. J. Infect. Dis..

[8] Moxon C.A., Heyderman R.S., Wassmer S.C. (2009). Dysregulation of coagulation in cerebral malaria. Mol. Biochem. Parasitol..

[9] Avril M., Bernabeu M., Benjamin M., Brazier A.J., Smith J.D. (2016). Interaction between Endothelial Protein C Receptor and Intercellular Adhesion Molecule 1 to Mediate Binding of *Plasmodium falciparum*-Infected Erythrocytes to Endothelial Cells. mBio.

[10] Brown H.C., Chau T.T., Mai N.T., Day N.P., Sinh D.X., White N.J., Hien T.T., Farrar J., Turner G.D. (2000). Blood-brain barrier function in cerebral malaria and CNS infections in Vietnam. Neurology.

[11] Turner L., Lavstsen T., Berger S.S., Wang C.W., Petersen J.E., Avril M., Brazier A.J., Freeth J., Jespersen J.S., Nielsen M.A. (2013). Severe malaria is associated with parasite binding to endothelial protein C receptor. Nature.

[12] Mohan Rao L.V., Esmon C.T., Pendurthi U.R. (2014). Endothelial cell protein C receptor: A multiliganded and multifunctional receptor. Blood.

[13] Pendurthi U.R., Rao L.V.M. (2018). Endothelial cell protein C receptor-dependent signaling. Curr. Opin. Hematol..

[14] Thanabalasundaram G., Schneidewind J., Pieper C., Galla H.J. (2011). The impact of pericytes on the blood-brain barrier integrity depends critically on the pericyte differentiation stage. Int. J. Biochem. Cell. Biol..

[15] Cavaglia M., Dombrowski S.M., Drazba J., Vasanji A., Bokesch P.M., Janigro D. (2001). Regional variation in brain capillary density and vascular response to ischemia. Brain Res..

[16] Nonaka H., Akima M., Hatori T., Nagayama T., Zhang Z., Ihara F. (2003). Microvasculature of the human cerebral white matter: Arteries of the deep white matter. Neuropathology.

[17] Chi J.T., Chang H.Y., Haraldsen G., Jahnsen F.L., Troyanskaya O.G., Chang D.S., Wang Z., Rockson S.G., van de Rijn M., Botstein D. (2003). Endothelial cell diversity revealed by global expression profiling. Proc. Natl. Acad. Sci. USA.

[18] Ge S., Pachter J.S. (2006). Isolation and culture of microvascular endothelial cells from murine spinal cord. J. Neuroimmunol..

[19] Saubamea B., Cochois-Guegan V., Cisternino S., Scherrmann J.M. (2012). Heterogeneity in the rat brain vasculature revealed by quantitative confocal analysis of endothelial barrier antigen and P-glycoprotein expression. J. Cereb. Blood Flow. Metab..

[20] Ball H.J., McParland B., Driussi C., Hunt N.H. (2002). Isolating vessels from the mouse brain for gene expression analysis using laser capture microdissection. Brain Res. Brain Res. Protoc..

[21] Fonta C., Barone P., Rodriguez Martinez L., Negyessy L. (2015). Rediscovering TNAP in the Brain: A Major Role in Regulating the Function and Development of the Cerebral Cortex. Subcell. Biochem..

[22] Calhau C., Martel F., Pinheiro-Silva S., Pinheiro H., Soares-da-Silva P., Hipolito-Reis C., Azevedo I. (2002). Modulation of insulin transport in rat brain microvessel endothelial cells by an ecto-phosphatase activity. J. Cell. Biochem..

[23] Laszik Z., Mitro A., Taylor F.B., Ferrell G., Esmon C.T. (1997). Human protein C receptor is present primarily on endothelium of large blood vessels: Implications for the control of the protein C pathway. Circulation.

[24] Hase Y., Ding R., Harrison G., Hawthorne E., King A., Gettings S., Platten C., Stevenson W., Craggs L.J.L., Kalaria R.N. (2019). White matter capillaries in vascular and neurodegenerative dementias. Acta Neuropathol. Commun..

[25] Moxon C.A., Wassmer S.C., Milner D.A., Chisala N.V., Taylor T.E., Seydel K.B., Molyneux M.E., Faragher B., Esmon C.T., Downey C. (2013). Loss of endothelial protein C receptors links coagulation and inflammation to parasite sequestration in cerebral malaria in African children. Blood.

[26] Alber J., Alladi S., Bae H.J., Barton D.A., Beckett L.A., Bell J.M., Berman S.E., Biessels G.J., Black S.E., Bos I. (2019). White matter hyperintensities in vascular contributions to cognitive impairment and dementia (VCID): Knowledge gaps and opportunities. Alzheimers Dement..

[27] Lin J., Wang D., Lan L., Fan Y. (2017). Multiple Factors Involved in the Pathogenesis of White Matter Lesions. Biomed. Res. Int..

[28] Weksler B.B., Subileau E.A., Perriere N., Charneau P., Holloway K., Leveque M., Tricoire-Leignel H., Nicotra A., Bourdoulous S., Turowski P. (2005). Blood-brain barrier-specific properties of a human adult brain endothelial cell line. FASEB J..

[29] Jung M., Kramer E., Grzenkowski M., Tang K., Blakemore W., Aguzzi A., Khazaie K., Chlichlia K., von Blankenfeld G., Kettenmann H. (1995). Lines of murine oligodendroglial precursor cells immortalized by an activated neu tyrosine kinase show distinct degrees of interaction with axons in vitro and in vivo. Eur. J. Neurosci..

[30] Weksler B., Romero I.A., Couraud P.O. (2013). The hCMEC/D3 cell line as a model of the human blood brain barrier. Fluids Barriers CNS.

[31] Kramer-Albers E.M., Bretz N., Tenzer S., Winterstein C., Mobius W., Berger H., Nave K.A., Schild H., Trotter J. (2007). Oligodendrocytes secrete exosomes containing major myelin and stress-protective proteins: Trophic support for axons?. Proteom. Clin. Appl..

[32] Stins M.F., Prasadarao N.V., Zhou J., Arditi M., Kim K.S. (1997). Bovine brain microvascular endothelial cells transfected with SV40-large T antigen: Development of an immortalized cell line to study pathophysiology of CNS disease. In Vitro Cell. Dev. Biol. Anim..

[33] Stins M.F., Pearce D., Choi H., Di Cello F., Pardo C.A., Kim K.S. (2004). CD4 and chemokine receptors on human brain microvascular endothelial cells, implications for human immunodeficiency virus type 1 pathogenesis. Endothelium.

[34] Tripathi A.K., Sullivan D.J., Stins M.F. (2006). *Plasmodium falciparum*-infected erythrocytes increase intercellular adhesion molecule 1 expression on brain endothelium through NF-κB. Infect. Immun..

